# Matrix Metalloproteinase-9: Its Interplay with Angiogenic Factors in Inflammatory Bowel Diseases

**DOI:** 10.1155/2014/643645

**Published:** 2014-03-31

**Authors:** Malgorzata Matusiewicz, Katarzyna Neubauer, Magdalena Mierzchala-Pasierb, Andrzej Gamian, Malgorzata Krzystek-Korpacka

**Affiliations:** ^1^Department of Medical Biochemistry, Wroclaw Medical University, Chalubinskiego 10, 50-358 Wroclaw, Poland; ^2^Department of Gastroenterology and Hepatology, Wroclaw Medical University, Borowska 213, 50-556 Wroclaw, Poland; ^3^Institute of Immunology and Experimental Therapy, Polish Academy of Sciences, Weigla 12, 53-114 Wroclaw, Poland

## Abstract

Matrix metalloproteinase- (MMP-) 9 is one of the main metalloproteinases reported to be involved in extracellular matrix degradation and recently also in triggering of angiogenic switch in the course of inflammatory bowel diseases (IBD). The goal of our studies was to estimate in one experimental setting the levels of MMP-9 in sera of Crohn's Disease (CD) and ulcerative colitis (UC) patients and to evaluate its possible diagnostic potential in comparison with other biochemical markers and selected proinflammatory and angiogenic factors. The study group included 176 subjects (CD = 64, UC = 85, control = 27). Concentrations of serum MMP-9 were significantly higher in active than inactive forms of IBD, being higher in active UC than in active CD. Both in the case of CD and UC serum MMP-9 positively correlated with disease activity, IL-6 levels, platelet and leukocyte count, midkine, and PDGF-BB, as well as in UC with ESR and in CD with CRP, IL-1, and VEGF-A. Diagnostic accuracy of MMP-9 in distinguishing active UC from active CD was 66%, and displayed higher specificity than CRP (79.0% versus 61.6%, resp.). Evaluation of serum MMP-9 concentrations could aid in differentiation of active UC from active CD. MMP-9 correlated better with inflammatory and angiogenic parameters in CD than in UC.

## 1. Introduction

Matrix metalloproteinases (MMPs) are a group of enzymes engaged in the degradation and remodeling of extracellular matrix (ECM). Nowadays six groups of these enzymes have been distinguished (collagenases, gelatinases, stromelysins, matrilysins, membrane-type, and a sixth group encompassing several other MMPs not classified in the previous categories), differing in structure, cellular localization, and substrate specificity [1]. Since these enzymes are involved in connective tissue remodeling occurring in the course of morphogenetic processes, therefore, they are a subject of a very strict regulation, which is executed, among others, by the expression of their specific inhibitors—tissue inhibitors of metalloproteinases (TIMPs) [1, 2]. TIMPs interact with MMPs on the 1 : 1 ratio, and any imbalance of this equilibrium as well as disturbances in the synthesis/degradation balance cause an excessive degradation of ECM or an excessive accumulation of connective tissue elements, which in consequence leads to pathological processes [2].

Inflammatory bowel diseases (IBD) belong to the diseases whose incidence is dramatically increasing in the last decades [3–5]. IBD encompasses three types of diseases: Crohn's disease (CD), ulcerative colitis (UC), and inflammatory bowel diseases undefined (IBDU). Among factors responsible for the development of IBD are genetic, microbiological, environmental, and immunological factors [6]. Recently also angiogenesis has been recognized as an important event in IBD development [7]. The involvement of MMPs in inflammatory processes has been documented both in animal models with experimentally induced IBD and in intestinal cell lines as well as in cultures of inflammatory altered tissues [8–10]. This involvement has been confirmed by histological studies, which demonstrated correlation between the expression of certain MMPs in tissue specimens from IBD patients and the degree of inflammation [11–13]. MMP-9 has been demonstrated to be the main metalloproteinase implicated in the development of IBD [8, 14]. Studies on MMP-9 deficient mice suggest that MMP-9 is involved already in the early stage of IBD development [8]. It has been demonstrated that it is engaged in diminishing cell adhesion and in the attraction of neutrophils to the site of injury [8, 15–17]. However, recent studies suggest that it is epithelial-derived and not neutrophil-derived MMP-9 that is responsible for the penetration of inflammatory cells into inflamed tissue [8, 16]. Furthermore, studies on cell lines and animal models have indicated that IBD development can be diminished by the application of metalloproteinases' inhibitors [14, 15, 18]. However, despite the growing body of evidence on the involvement of MMPs in IBD, there is only limited number of studies which would try to relate the changes observed on the tissue level to the systemic concentrations in body fluids such as urine or blood [19–24]. The demonstration that the changes of MMPs on the organ level are reflected by their concentration or activity in easily accessible biological material would aid in the diagnosis and differentiation and monitoring of the course, as well as effectiveness of IBD treatment.

In our previous study, we have already demonstrated that in pediatric patients serum concentrations of MMP-9 correlate with indices of inflammation and reflect severity of Crohn's disease [22]. The goal of our present studies was to estimate the levels of MMP-9 in the serum of patients with CD and UC and to evaluate its possible potential in diagnostics and differentiation of IBD as well as to compare it to other biochemical markers or parameters used in connection with this disease, including selected angiogenic factors.

## 2. Materials and Methods

The study group comprised 149 patients with recognized IBD, aged from 18 to 79 years (mean age 47.7), hospitalized in the Department of Gastroenterology and Hepatology, Wroclaw Medical University, Wroclaw, Poland, in the years 2005–2008 due to disease flare or admitted for control examination. Patients with the coexistence of other severe systemic diseases, malignancies, liver diseases, or pregnancies were not included. Among patients enrolled into the study, 38 (19 males and 19 females) had active Crohn's disease (CDa) (mean age 38.0, range 20–67), 26 (13 males and 13 females) inactive Crohn's disease (CDi) (mean age 40.8, range 19–69), 38 (20 males and 18 females) active ulcerative colitis (UCa) (mean age 43.1, range 18–79), and 47 (28 males and 19 females) inactive ulcerative colitis (UCi) (mean age 48.7, range 18–76). Disease activity was estimated on the basis of Crohn's Disease Activity Index (CDAI) in the case of CD and Clinical Activity Index (CAI), also known as Rachmilewitz Index (RI), in the case of UC. CDAI is based on the evaluation of vital parameters, clinical findings, and medical history [25]. CAI encompasses stool frequency, number of stools with blood, general well-being, abdominal pain/cramp, fever, extraintestinal manifestations, and laboratory tests: erythrocyte sedimentation rate (ESR) and hemoglobin (HGB) concentration [26]. None of the patients included into a study received biological treatment.

Control group consisted of 27 (12 males and 15 females) healthy volunteers and patients aged from 18 to 66 years (mean age 26.6) hospitalized in the Clinic, in whom organic diseases as well as metabolic disorders were excluded on the basis of diagnostic procedures. The patients included into the control group were diagnosed with functional disorders such as functional dyspepsia, irritable bowel syndrome, and functional constipation.

Blood samples were drawn by venous puncture in a fasting state. Sera were obtained from clotted (30 min, room temperature) and centrifuged (15 min, 1500 ×g) blood. Serum samples were stored at −80°C until analysis.

MMP-9 concentrations were estimated by an enzyme double-antibody indirect immunoassays with DuoSet Human MMP-9/TIMP-1 Complex provided by R&D Systems (Minneapolis, MN, USA). Circulating interleukins 1 and 6 (IL-1 and IL-6), tumor necrosis factor-*α* (TNF-*α*), and vascular endothelial growth factor A (VEGF-A) were determined by an enzyme double-antibody indirect immunoassays using, respectively, PeliKine Compact human IL-1, IL-6, TNF-*α* (Sanquin, Amsterdam, Holland), and eBioscence VEGF-A (Vienna, Austria) ELISA kits. All determinations were conducted in accordance with manufacturers' protocols.

Serum high-sensitive CRP was determined by the latex particle-enhanced immunoturbidimetric method with the CRPex-HS CRP test (Good Biotech Corp., Taichung, Taiwan), with protein multicalibrator (ProDia International, Sharjah, UAE).

Data on PDGF-BB (Strathmann Biotec GmbH, Hamburg, Germany) and midkine (Biovendor, Czech Republic) for the purposes of correlation analysis were retrieved from our previous studies [27–29]. Data on HGB, platelets count (PLT), leukocyte count (WBC), and ESR were retrieved from patients' medical records.

### 2.1. Statistical Analysis

After log-transformation serum concentrations of MMP-9 had a skewed distribution as analyzed with D'Agostino-Pearson test for normality. Therefore, the average concentrations of MMP-9 are presented as median values accompanied by 95% confidence interval (95% CI). The significance of differences between groups was examined with Kruskal-Wallis test. Correlation analysis was conducted with Spearman test. Differences in incidence rates were analyzed with Fisher's exact test. All tests were two-sided and *P* values ≤0.05 were considered significant. Due to scarcity of serum samples or incomplete information in the patients' medical records, correlation analysis was not conducted in all the patients enrolled; the number of patients lacking examination, however, was negligible.

The diagnostic values of MMP-9 and CRP were evaluated using receiver operating characteristic (ROC) curves analysis. The overall performance was expressed as the area under ROC curve (AUC) with 95% CI and* p*-statistics for the difference between calculated AUC and AUC = 0.5 (marker without discriminative power). Cut-off values corresponding to the highest accuracy were determined and the related sensitivities and specificities together with likelihood ratios for positive and negative results (+LR and −LR) were calculated. Youden index (sensitivity + specificity − 1), a summary measure, was calculated as well. Statistical analysis was conducted with MedCalc version 11.4.4.0 statistical software.

## 3. Ethical Considerations

The study protocol was approved by the Medical Ethics Committee of Wroclaw Medical University, Wroclaw, Poland, and the study was conducted in accordance with the Helsinki Declaration of 1975, as revised in 1983.

## 4. Results

The parameters of study population are presented in Table 1.

Statistical analysis indicated that the studied groups (CD, UC, and control group) did not differ in terms of gender distribution (*P* = 0.498). There were statistical differences in age of participants in particular groups (*P* < 0.001); however, MMP-9 concentration did not depend on age (rho = 0.0729, *P* = 0.341).

MMP-9 concentrations in sera of patients with CD were significantly lower than those with UC (*P* = 0.016), but did not differ from control samples (*P* = 0.086). However, when active form of CD was taken into consideration, MMP-9 concentrations were significantly higher than in control group (*P* = 0.003) as well as in patients with inactive CD (*P* < 0.001).

In the case of UC, MMP-9 concentrations in patients with UC were significantly higher than those in control group (*P* < 0.001). Moreover, MMP-9 concentrations in UCa were statistically higher (*P* < 0.0001) not only than concentrations in control group but also than concentrations in UCi, CDi, and CDa. Additionally, MMP-9 concentrations in UCi were higher than those in CDi (*P* = 0.047).

There were no differences in MMP-9 concentrations between groups with inactive form of the disease and control group (CDi: *P* = 0.593, UCi: *P* = 0.102, resp.).

**Figure 1:**
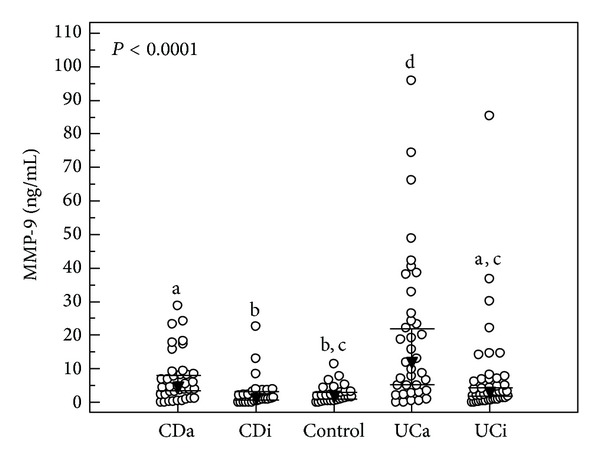
The distribution of serum MMP-9 concentrations in the studied groups of IBD. CDa: active Crohn's disease; CDi: inactive Crohn's disease; UCa: active ulcerative colitis; UCi: inactive ulcerative colitis. Open circles represent MMP-9 serum concentration of individual subjects. Dark triangles represent median values accompanied by 95% confidence interval represented as horizontal bars. The groups with the same indices do not differ statistically.

Figure 1 presents the distribution of MMP-9 concentrations in particular groups.

**Figure 2:**
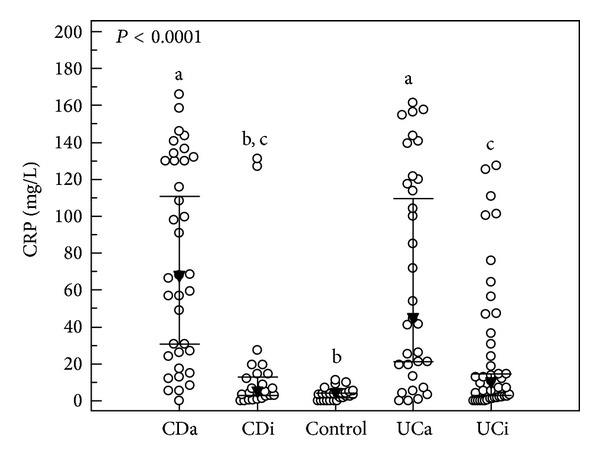
The distribution of serum CRP concentrations in the studied groups of IBD. CDa: active Crohn's disease; CDi: inactive Crohn's disease; UCa: active ulcerative colitis; UCi: inactive ulcerative colitis. Open circles represent CRP serum concentration of individual subjects. Dark triangles represent median values accompanied by 95% confidence interval represented as horizontal bars. The groups with the same indices do not differ statistically.

Similar analysis for CRP (Table 1 and Figure 2) indicated statistically significant differences between CD, UC and control groups (*P* < 0.0001). However, there were no differences between CRP levels in CD and UC patients (*P* = 0.231). When inactive and active forms were taken into consideration, CRP in control group was significantly lower (*P* < 0.0001) than this parameter in the case of CDa, UCi, and UCa. CRP levels in UCa and CDa did not differ statistically. There were also no differences between CDi and UCi in respect of this parameter (Figure 2).

### 4.1. Correlations between MMP-9 Concentrations and Indices of IBD and Angiogenic Factors

Correlation between MMP-9 concentrations and angiogenic factors in CD and UC are presented in Table 2. In the case of CD, MMP-9 positively correlated with CDAI, CRP, IL-1, IL-6, PLT, WBC, midkine, VEGF A, and PDGF-BB. In the case of UC significant correlations were found between MMP-9 concentrations and CAI, ESR, IL-6, PLT, WBC, midkine, and PDGF-BB and they were weaker than for CD. When active and inactive forms of the disease were taken into consideration, correlation between MMP-9 and CRP was sustained only in case of CDa (rho = 0.463, *P* = 0.004), but not in case of CDi (rho = 0.281, *P* = 0.183). Similarly, as when a whole cohort of UC patients was taken into consideration, there was also no correlation between MMP-9 and CRP in the case of either UCa (rho = 0.130, *P* = 0.471) or UCi (rho = 0.08, *P* = 0.609). Also in case of control group no correlation was found between MMP-9 and CRP (rho = 0.026, *P* = 0.896).

We also determined whether any of the studied inflammatory or proangiogenic factors could be useful in differentiation between active forms of CD and UC. Similarly, as in the case of CRP, in our cohort of patients none of the studied parameters differed in the case of CDa and CDi (Table 3).

### 4.2. Diagnostic Performance of MMP-9 as Disease Marker

Diagnostic accuracy of MMP-9 in distinguishing CD from control group, evaluated as area under ROC curve, was 61% (59–72, *P* = 0.055). For comparison, diagnostic accuracy of CRP in this case was 84% (75–91, *P* < 0.0001). Optimal cut-off values, specificities, sensitivities, +LR, and −LR as well as Youden indices are presented in Table 4(a).

Diagnostic accuracy of MMP-9 in distinguishing UC from control group, was 72% (63–80, *P* < 0.0001). For comparison, diagnostic accuracy of CRP in this case was 78% (69–85, *P* < 0.0001). Optimal cut-off values, specificities, sensitivities, +LR, −LR as well as Youden indices are presented in Table 4(b).

Diagnostic accuracy of MMP-9 in distinguishing active UC from active CD was 66% (54–77, *P* = 0.012). For comparison, diagnostic accuracy of CRP in this case was 55% (49–67, *P* = 0.482). Optimal cut-off values, specificities, sensitivities, +LR, and −LR as well as Youden indices are presented in Table 4(c).

## 5. Discussion

Early diagnosis and differentiation between Crohn's disease and ulcerative colitis are vital for appropriate diagnostic strategy and prediction of the disease course. However, none of the routinely used biochemical markers such as CRP or ESR display sufficient specificity to serve as the “gold standard” test. Therefore, intensive studies in this area are in progress.

Expression of metalloproteinases is altered in diseases with inflammatory background [12, 13, 30]. Many studies point to MMP-9 as a key enzyme engaged in the degradation of alimentary tract tissues in the course of IBD [8, 14, 15, 31–33]. MMP-9 is also involved in the shedding and activation of biologically active molecules, which further perpetuates pathological processes observed in IBD [33] and renders MMP-9 an interesting diagnostic and therapeutic target. Manfredi et al. [20] studied the usefulness of urinary metalloproteinases in evaluation of pediatric IBD patients and found that urinary MMP-2 and MMP-9 complexed with neutrophil gelatinase associated lipocalin were independent predictors of CD and UC. Corroborating their immunohistochemical observations [34], Lakatos et al. noted that serum concentrations of MMP-9 were higher in both UC and CD patients compared to controls and correlated well with the disease activity [23]. Our results, obtained on a larger cohort of patients, confirmed significant elevation of serum MMP-9 in UC but only a tendency towards such elevation in CD. Yet, when analyzing active and inactive forms of IBD, we noted that MMP-9 concentrations in active CD and UC were significantly higher not only than those detected in controls but also with respect to concentrations measured in patients with inactive forms.

To the best of our knowledge, this is the first report demonstrating that MMP-9 concentrations in active UC are significantly higher not only than those in controls and inactive UC but also than those in active CD. None of the other examined factors, such as ESR, CRP, proinflammatory, and angiogenic factors, were able to discriminate between active CD and active UC. This finding might be of value when considering MMP-9 as a supportive marker in differential diagnosis of patients with active IBD helping in decision making concerning appropriate therapeutic strategies. When comparing ability of MMP-9 and CRP in distinguishing total CD and UC patients from controls, CRP exhibited better sensitivities and specificities, but in distinguishing between active UC and CD, MMP-9 had similar sensitivity but much higher specificity than CRP. It has been already proposed that urine MMPs may serve as a biomarker identifying active forms of IBD [20]. Manfredi et al. demonstrated that endoscopy confirmed active form of IBD in all patients in whom MMP-2 and/or MMP-9 were earlier detected in the urine samples, while clinical disease activity indices pointed to active disease only in 75% of those cases [20].

Many studies indicate that correlations between markers routinely used in the diagnostics of IBD and clinical activity of the disease are much stronger for CD than UC. Elevation of CRP is observed in 70%–100% of CD and only 50%–60% of UC patients [35]. In their recent studies, Karoui et al. pointed that CRP correlates well with clinical and endoscopic activity in UC, but alone is not sufficient to determine disease activity due to its low sensitivity [36]. Our results indicate that MMP-9, in combination with CRP or other markers, might be useful in differential diagnostics of IBD.

Recent studies point to angiogenesis as an important event in the development of IBD [7]. Metalloproteinases enhance the process by degrading ECM, releasing and processing proangiogenic factors from ECM and weakening cell to cell adhesion [37]. VEGF-A is the best characterized positive regulator of angiogenesis [38] and in animal models its release from ECM has been associated with upregulation of MMP-9 expression [39]. Accordingly, we observed a strong correlation between circulating VEGF-A and serum MMP-9 but only in CD patients. This seems to corroborate with the results of Konno et al. [40] who demonstrated that all factors in VEGF-Ets-1 cascade including VEGF receptors were upregulated in UC and downregulated in CD. This might imply that circulating levels of this cytokine would be decreased in UC. However, since the results on circulating levels of VEGF-A in UC and CD are not totally unequivocal—the majority [41–43], but not all of them [44], demonstrate elevated levels of this factor—it seems that this aspect requires further elucidation in a larger scale systematic research. This issue appears to be important especially in light of recent studies of Tolstanova et al. [45] who demonstrated concomitant upregulation of MMP-9, VEGF-A, PDGF-BB, and endostatin in rodent models of UC. They suggested that the increase in endostatin plays a protective role against increased level of VEGF in UC and that MMP-9, which generates endostatin, is a key enzyme responsible for the balance between those two cytokines.

Similar pattern was observed for midkine, a novel cytokine with proangiogenic and proinflammatory functions, systemic changes of which have also been noted in IBD [28, 29]. Correlation between MMP-9 and this proangiogenic growth factor was again stronger in the case of CD than UC. The detailed relation between midkine and MMPs requires further investigation, however, it is known that midkine, similarly to MMP-9 can up-regulate VEGF-A synthesis [46].

MMP-9 has also been suggested to participate in the recruitment of pericytes to new vessels [47], the process in which a major role is played by PDGF-BB [48]. We have already demonstrated that circulating PDGF-BB was elevated in active CD and UC [27]. Presently, we found that its concentration moderately correlated with MMP-9 in both CD and UC, which is in agreement with the fact that no differences in PDGF-BB between CD and UC either on tissue or systemic level have been noted [27, 40]. Availability of PDGF-BB is regulated by its binding to ECM components and its release requires proteolytic removal of C-terminal retention motif; however, factors responsible for this process are unknown [49]. It can be speculated that MMP-9 might participate either in extracellular removal of the retention motif of PDGF-BB or in degradation of ECM elements involved in PDGF-BB binding which would allow PDGF to exert its biological functions.

Correlations with the studied proinflammatory parameters were stronger in the case of CD than UC as well, indicating that MMP-9 more profoundly reflects inflammatory process in the case of CD. This is in agreement with the results of Gross et al. [50], who noted stronger elevation of IL-6 in CD than in UC and also with the recent findings on the association of Th17 cells with inflammatory process in CD [51].

Numerous aspects of MMP-9 engagement in the development of IBD aroused interest in its possible therapeutic application. Studies conducted on explant cultures as well as on animal models demonstrated that synthetic inhibitors of MMPs can downregulate MMP-9 levels and at the same time inhibit mucosal damage and improve morphological and histopathological scores [10, 24, 31]. Attempts with inhibitors of MMPs have already been successful in a treatment of periodontitis [52]; therefore, there is a rational possibility that selective inhibitors of MMP-9 could find application also in the treatment of IBD.

## 6. Conclusions

Studies conducted on our cohort of patients demonstrated that determination of MMP-9 could be useful as a possible supportive marker permitting differentiation between active and inactive forms of IBD as well as between active UC and active CD. Stronger correlations of MMP-9 with inflammatory indices and angiogenic factors in the case of CD in respect to patients with UC might arise from diverse molecular backgrounds of these diseases.

## Figures and Tables

**Table 1:** Characteristics of study population.

Index	Controls	UC			CD		
		All	UCa	UCi	All	CDa	CDi
MMP-9 [ng/mL]	1.91 (0.83–3.03)	5.02 (3.22–7.68)	11.93 (5.2–22.0)	2.99 (1.76–4.36)	3.47 (2.12–4.08)	4.61 (3.48–7.96)	1.22 (0.61–3.12)
CRP [mg/L]	3.7 (1.8–4.0)	18.8 (10.4–31.9)	44.5 (21.0–109.7)	9.5 (3.2–14.3)	25.1 (12.8–57.1)	67.3 (30.5–111.0)	17.3 (2.4–32.1)
CDAI	—	—	—	—	160 (129–205)	227 (186–259)	94 (52–114)
CAI	—	3 (2–4)	5 (4–7)	1 (0–2)	—	—	—
ESR [mm/h]	ND	16 (12–27)	36.5 (17–50)	11.5 (8–19)	20 (16–26)	31 (20–42)	13 (10–17)
HGB [g/dL]	ND	12.8 (12.1–13.5)	11.7 (10.4–12.6)	13.5 (12.8–14.0)	12.2 (11.7–13.0)	11.7 (10.9–12.4)	13.0 (12.2–13.9)
PLT [×10^9^/L]	ND	299 (277–324)	349 (304–416)	273 (252–292)	345 (253–397)	389 (334–464)	247 (208–370)
WBC [×10^9^/L]	ND	7.3 (6.2–8.1)	8.4 (6.6–9.9)	6.6 (6.0–7.4)	6.8 (5.7–8.2)	7.7 (5.6–8.8)	6.2 (5.2–7.9)

If not otherwise stated, data are presented as medians with 95% CI around median; CD: Crohn's disease; CDa: active Crohn's disease; CDi: inactive Crohn's disease; UC: ulcerative colitis; UCa: active ulcerative colitis; UCi: inactive ulcerative colitis; M/F: males/females ratio; CRP: C-reactive protein; CDAI: Crohn's disease activity index; CAI: clinical activity index; ESR: erythrocyte sedimentation rate; HGB: haemoglobin; PLT: platelet count; WBC: white blood cell count, ND: no data.

**Table 2:** Correlations between MMP-9 and studied factors in Crohn's disease (CD) and ulcerative colitis (UC).

Factor	CD		UC	
	rho	*P*	rho	*P*
CRP	0.479	<0.0001*	0.201	0.075
ESR	0.194	0.139	0.394	<0.001*
HGB	−0.204	0.121	−0.209	0.062
IL-1	0.317	0.035*	−0.194	0.139
IL-6	0.532	<0.001*	0.277	0.038*
PLT	0.418	0.002*	0.248	0.026*
TNF-*α*	−0.0131	0.930	0.0233	0.859
WBC	0.476	<0.001*	0.266	0.018*
Midkine	0.516	<0.001*	0.228	0.037*
VEGF-A	0.377	0.040*	0.072	0.660
PDGF-BB	0.326	0.0408*	0.301	0.032*
CDAI	0.292	0.029*	—	—
CAI			0.342	0.010*

CRP: C-reactive protein; ESR: erythrocyte sedimentation rate; HGB: hemoglobin; IL-1, -6: interleukin-1, -6; PLT: platelet count; TNF-*α*: tumor necrosis factor *α*; WBC: white blood cells; VEGF-A: vascular endothelial growth factor A; PDGF-BB: platelet-derived growth factor-BB; CDAI: Crohn's disease activity index; CAI: clinical activity index. Asterisks indicate statistically significant correlations.

**Table 3:** Comparison of selected inflammatory and angiogenic factors between active Crohn's disease (CDa) and active ulcerative colitis (UCa).

Factor	CDa	UCa	*P* value
ESR [mm/h]	37 (28–45)	37 (27–47)	*P* = 0.912
IL-1 [ng/L]	0.98 (0.34–1.4)	0.88 (0.36–3.0)	*P* = 0.479
IL-6 [ng/L]	3.39 (2.0–5.7)	4.1 (2.4–5.1)	*P* = 0.661
TNF-*α* [ng/L]	0.39 (0.18–0.94)	0.53 (0.21–1.02)	*P* = 0.604
PDGF [*μ*g/L]	7.1 (5.5–8.6)	8.0 (5.0–11.5)	*P* = 0.449
Midkine [pg/mL]	375 (320–489)	348 (269–467)	*P* = 0.743
VEGF-A [ng/mL]	0.68 (0.61–0.96)	0.89 (0.54–1.33)	*P* = 0.855

If not otherwise stated, data are presented as medians with 95% CI around median.

**Table:** (a)

Marker	Cut-off value	Sensitivity	Specificity	+LR	−LR	Youden index
MMP-9	3.22 ng/mL	53.1%	77.8%	2.39	0.6	0.309
CRP	11.4 mg/L	66.7%	100%		0.33	0.667

**Table:** (b)

Marker	Cut-off value	Sensitivity	Specificity	+LR	−LR	Youden index
MMP-9	4.48 ng/mL	54.1%	85.2%	3.65	0.54	0.393
CRP	11.4 mg/L	60%	100%		0.4	0.600

**Table:** (c)

Marker	Cut-off value	Sensitivity	Specificity	+LR	−LR	Youden index
MMP-9	9.35 ng/mL	54.0%	79.0%	2.57	0.58	0.330
CRP	53.78 mg/L	54.5%	61.6%	1.4	0.74	0.157
